# Ribosome biogenesis: A central player in liver diseases

**DOI:** 10.1016/j.gendis.2025.101512

**Published:** 2025-01-04

**Authors:** Wei Luo, Jing Zhou, Yongmin Yan, Xuezhong Xu

**Affiliations:** a Changzhou Key Laboratory of Molecular Diagnostics and Precision Cancer Medicine, Wujin Hospital Affiliated with Jiangsu University (Wujin Clinical College of Xuzhou Medical University), Changzhou, Jiangsu 213017, China; b Wujin Institute of Molecular Diagnostics and Precision Cancer Medicine of Jiangsu University, Changzhou, Jiangsu 213017, China; c Department of Laboratory Medicine, Wujin Hospital Affiliated with Jiangsu University, Changzhou, Jiangsu 213017, China

**Keywords:** Hepatitis C virus, Hepatocellular carcinoma, Liver cirrhosis, Liver fibrosis, Liver regeneration, Nonalcoholic fatty liver disease, Ribosome

## Abstract

Ribosome biogenesis is a multi-step process that initiates within the nucleolus, terminates in the cytoplasm, and determines the rate of protein synthesis. Ribosome biogenesis is essential for maintaining liver function. In eukaryotes, it involves producing and assembling approximately 200 factors and 80 ribosomal proteins. Mutations in ribosome proteins, ribosomal RNA processing, and ribosome assembly factors in the liver can result in liver disease. Hepatitis C virus causes acute or chronic infection and liver disease, which can progress to liver cirrhosis, cancer, and death. This review provides an overview of the effects of ribosomal biogenesis, including ribosomal RNA, ribosomal proteins, and ribosome biogenesis factors, on liver regeneration, hepatitis C virus, nonalcoholic fatty liver disease, liver fibrosis, cirrhosis, and liver cancer. It lists drugs that exploit ribosome biogenesis to treat liver cancer. Targeting ribosome biogenesis shows promise as a therapeutic approach. A better understanding of this process will contribute to developing effective and targeted therapeutic strategies for ribosome biogenesis disorders.

## Introduction

The primary function of ribosomes is to synthesize proteins using mRNA as a template and amino acids as raw materials.^1^^,^^2^ Ribosomes impact the rate of protein synthesis and play a role in cell proliferation, differentiation, apoptosis, and transformation.^3^, ^4^, ^5^ The liver is an important hub for material and energy metabolism. Acute or chronic liver damage is usually caused by alcohol, drugs, and toxic compounds. If it is not effectively relieved, it can further deteriorate into hepatitis C virus (HCV), nonalcoholic fatty liver disease (NAFLD), liver fibrosis, cirrhosis, or liver cancer.^6^ Several studies have shown that ribosomes are involved in liver growth, disease, and cancer.^7^^,^^8^ In recent years, increasing evidence has linked ribosome biogenesis to the liver. This article aims to review the mechanism of ribosome progression in the liver and provide new insights into the relationship between ribosome biogenesis and the liver. Additionally, it explores potential therapeutic approaches based on the latest findings.

## Ribosome and its biogenesis

**Figure 1:**
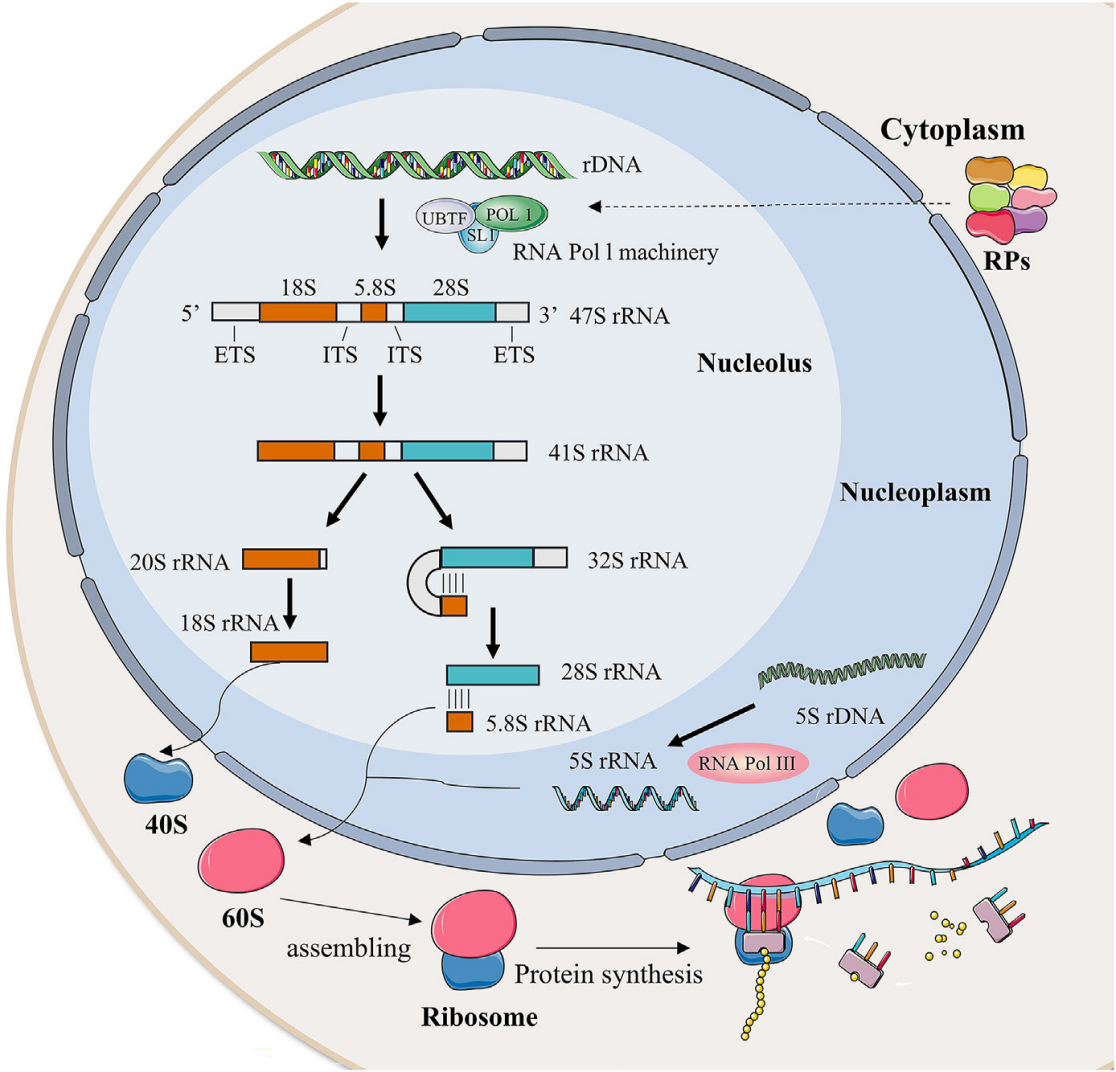
Schematic of ribosome biogenesis. Eukaryotic ribosome biogenesis involves RNA polymerases I/II/III (Pol I, Pol II, and Pol III), transcribing rDNA to rRNA, and producing 47S pre-rRNA in the nucleolus. Subsequently, the four rRNA molecules assemble with ribosomal proteins (RPs) to form a small ribosomal subunit (40S) and a large ribosomal subunit (60S). Following assembly, the ribosome complex is exported from the nucleolus to the cytoplasm, maturing into functional ribosomes for protein synthesis.

The ribosome is crucial for protein synthesis within a cell. The process of ribosome biogenesis involves the creation of ribosomes, which are made up of ribosomal RNAs and ribosomal proteins (RPs).^9^, ^10^, ^11^, ^12^ This complex and dynamic process begins in the nucleolus. It consists of three main stages: ribosomal DNA (rDNA) transcription into precursor ribosomal RNA (pre-rRNA), post-transcriptional processing from pre-rRNA to mature rRNA, and ribosome assembly.^13^^,^^14^ In eukaryotes, ribosome biogenesis requires the coordinated production of over 200 ribosome assembly factors (Fig. 1).^15^, ^16^, ^17^

Eukaryotic ribosomes comprise the small (40S) and large (60S) subunits. The biogenesis of ribosomes begins with the transcription of the 47S rDNA into 47S pre-rRNA. This transcription is carried out by RNA polymerase I (RNA Pol I) and is coordinated by selectivity factor 1 (SL1), upstream binding factor (UBF), transcription initiation factor 1 (TIF-1A), and transcription termination factor 1 (TTF-1).^18^^,^^19^ This is the critical rate-limiting step in ribosome biogenesis.^20^, ^21^, ^22^ Numerous RPs, non-ribosomal proteins, and small nucleolar RNAs (snoRNAs) are involved in the processing of ribosomal RNA and participate in the splicing process of ribosomal RNA (rRNA).^23^, ^24^, ^25^, ^26^, ^27^ The 5S rRNA is transcribed by RNA polymerase III (RNA Pol III) in the nucleoplasm and then transported to the nucleolar region for assembly.^28^, ^29^, ^30^, ^31^, ^32^ Conversely, the RP-encoding gene is transcribed by RNA polymerase II (RNA Pol II) in the nucleoplasm.^33^, ^34^, ^35^, ^36^ The 47S rRNA rapidly combines with RPs to form a ribonucleoprotein complex, undergoes multiple cleavages, and is eventually cleaved into 28S rRNA, 18S rRNA, and 5.8S rRNA.^37^, ^38^, ^39^

Cleavage and methylation are essential processes in pre-rRNA maturation into mature rRNA. During this process, more than 100 nucleotides in the 47S pre-rRNA are methylated and then cleavage into multiple RNA strands through a series of enzymatic reactions. The 47S rRNA is cleaved at the 5′ external transcriptional spacers (ETS) by a nuclease, resulting in the formation of 41S pre-rRNA and the release of 32S and 20S pre-rRNA. The internal transcriptional spacer (ITS) is also cleaved to produce mature 28S rRNA, 18S rRNA, and 5.8S rRNA.^40^, ^41^, ^42^ Methylation modification of rRNA occurs with the involvement of a complex formed by snoRNA and ribonucleoprotein.^43^, ^44^, ^45^

RPs are categorized into large ribosomal subunit proteins (RPL) and small ribosomal subunit proteins (RPs).^46^^,^^47^ They are transcribed in the nucleoplasm, translated into the cytoplasm, and then imported into the nucleolus, where they assemble with their respective ribosomal subunits.^48^ The 18S rRNA binds to 33 ribosomal small subunit proteins, forming the 40S small subunit. The 28S rRNA, 5.8S rRNA, and 5S rRNA combine with 46 ribosomal large subunit proteins to create the 60S large subunit.^49^^,^^50^ The ribosomal subunits are transported to the cytoplasm for final maturation.^51^ To ensure efficient export of subunits, export receptors interact with the hydrophobic central channel of the nuclear pore complex.^52^, ^53^, ^54^ In the cytoplasm, further rRNA folding, processing, assembly, and release of transport factors contribute to ribosomal subunit maturation.^55^, ^56^, ^57^, ^58^ Once fully mature, both ribosomal subunits can participate in protein translation in the cytoplasm.

## Ribosome biogenesis: a key regulator of liver function

**Figure 2:**
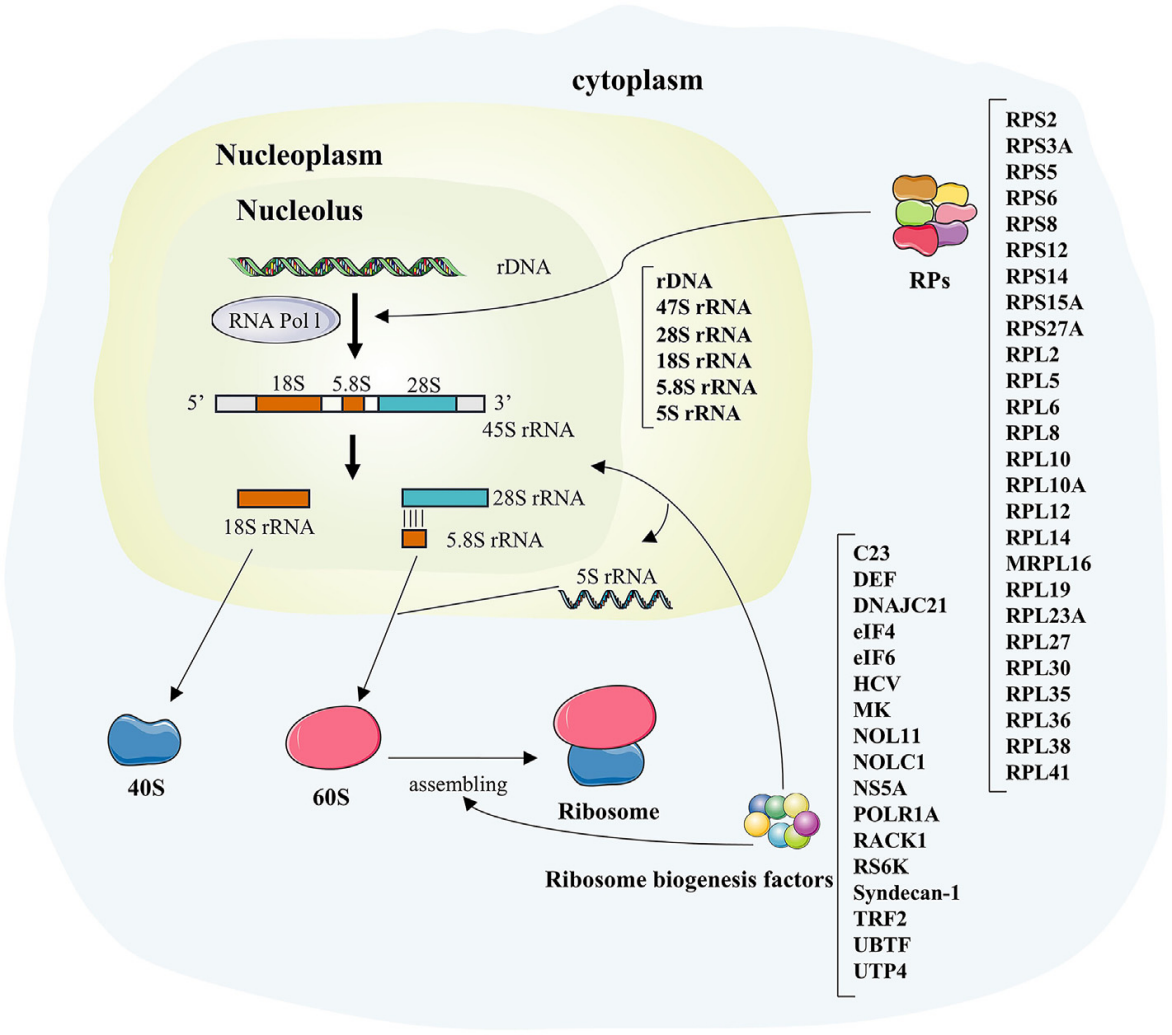
Schematic of the involvement of various rDNA, rRNA, ribosomal proteins (RPs), and ribosome biogenesis factors in ribosome biogenesis in the human liver. RPs act in rDNA transcription to generate rRNA. Ribosome biogenesis factors act on the cleavage of the precursor 45S rRNA into rRNA and the assembly of the 40S small subunit and the 60S large subunit into ribosomes.

Ribosome biogenesis has been linked to several human diseases, including Diamond-Blackfan anemia,^59^^,^^60^ brachycephaly,^61^ and spondyloepimetaphyseal dysplasia.^62^ Additionally, it is involved in the development of various types of human cancers, such as lung cancer, gastric cancer, liver cancer, colorectal cancer, and glioblastoma.^63^, ^64^, ^65^, ^66^ The liver is one of the most crucial organs within the human body, being important for functions such as protein synthesis and lipid/drug metabolism.^67^ Chronic hepatitis triggers the proliferation and remodeling of extracellular matrix such as collagen, leading to liver fibrosis and cirrhosis, and further develops into liver cancer.^68^^,^^69^ Understanding ribosome biogenesis in the liver may lead to the development of new strategies to treat liver disease (Fig. 2).

## Ribosome biogenesis and liver regeneration

**Figure 3:**
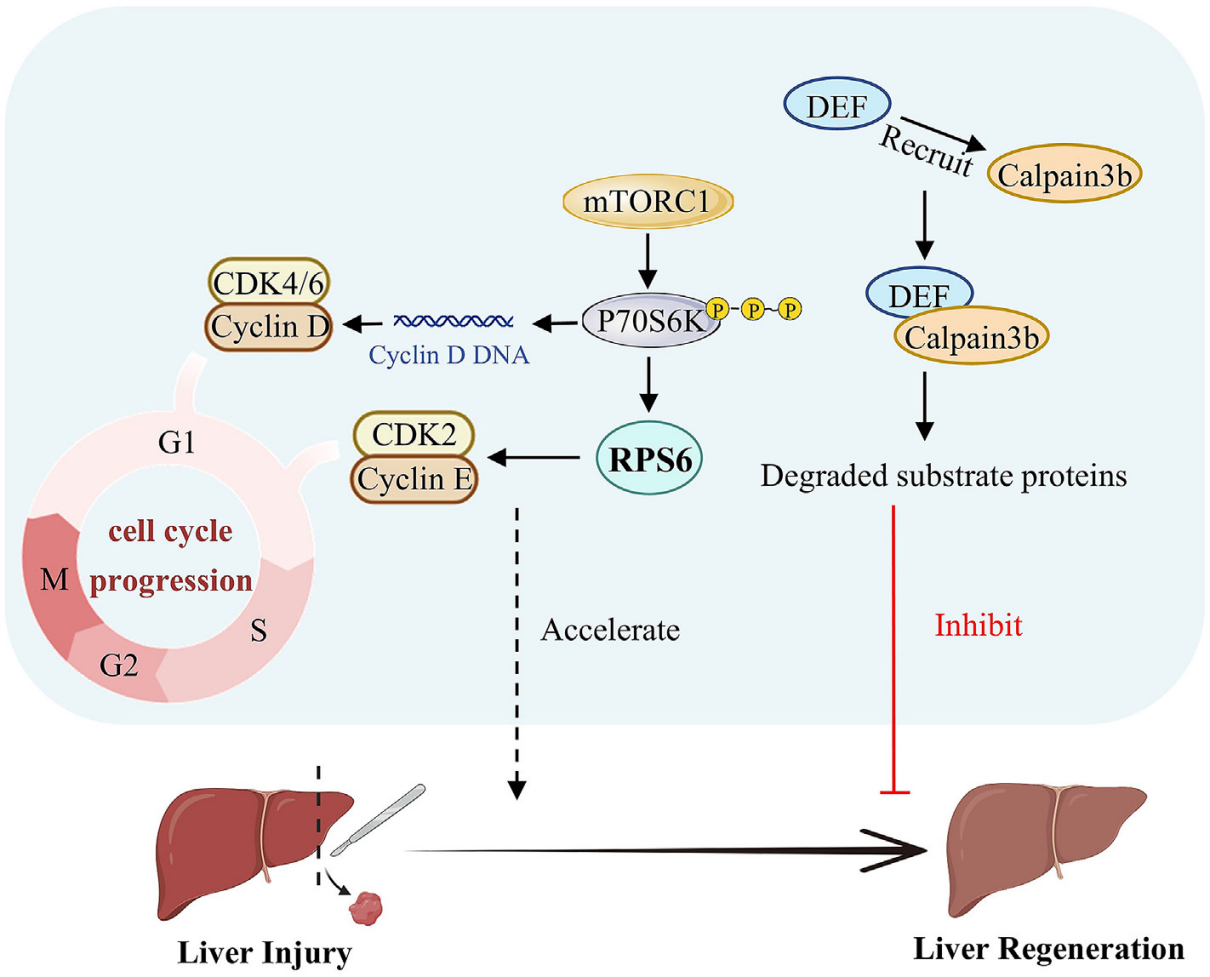
Schematic of liver regeneration after liver injury promoted by ribosomal proteins. P70S6K promotes cyclin D DNA replication and cyclin D protein synthesis and RPS6 promotes cyclin E expression, activating cell cycle progression, and promoting liver regeneration. DEF/UTP25 recruits Calpain3b to degrade protein substrates, and Def-Capn3 leads to protein accumulation in the nucleolus, causing nuclear vacuolization and impaired liver regeneration in mice.

Liver regeneration is a crucial biological process after liver injury. Ribosome biogenesis is critical in maintaining liver homeostasis and promoting regeneration (Fig. 3).^70^^,^^71^ Studies have shown that during liver regeneration in rats, there is a substantial increase in ribosomes in the cytoplasm of hepatocytes. Inhibition of ribosome biogenesis has been found to reduce the liver regeneration rate.^72^, ^73^, ^74^ KEGG pathway analysis of the termination stage of liver regeneration after partial hepatectomy in mice, using iTRAQ, revealed significant up-regulation of the “ribosome” pathways. In contrast, “metabolic pathways” were significantly down-regulated. These findings suggest that the termination phase of liver regeneration primarily focuses on restoring cellular structure and function.^75^

During rat liver regeneration, increased rRNA synthesis activates the mechanisms responsible for rRNA transcription in the central fibers, forming a distinct pattern known as dense fibrillar component by binding to ribonucleoprotein transcripts.^76^^,^^77^ Following partial liver resection, enhanced phosphorylation of P70S6 kinase (P70S6K) through the activation of phosphoinositide 3-kinase (PI3K)/mechanistic target of rapamycin (mTOR) pathway promotes cyclin D DNA replication and cyclin D protein synthesis, thereby promoting hepatocyte proliferation.^78^^,^^79^ The 40S small subunit synthetic protein RPS6, is a substrate of P70S6K and promotes cyclin E expression, activating cell cycle progression and promoting liver regeneration.^80^^,^^81^ DEF/UTP25, a nucleolar protein involved in ribosome biogenesis, localizes to the nucleolus by recruiting Calpain3b to degrade protein substrates. Disruption of Def-Capn3 leads to protein accumulation in the nucleolus, causing nuclear vacuolization and impaired liver regeneration in mice.^82^ Increased ribosome biogenesis is beneficial to liver regeneration after liver injury.

## Ribosome biogenesis and HCV

HCV is one of the important pathogens causing chronic liver infections in humans. Chronic HCV infection can lead to a series of liver lesions, such as hepatic steatosis, inflammation, fibrosis, cirrhosis, and primary liver cancer.^83^ Differential co-expression analysis of liver gene expression in samples from HCV cirrhotic patients showed that these differentially expressed genes may be involved in hepatocellular carcinoma (HCC) through the ribosome pathway and serve as potential therapeutic targets for the treatment of HCC.^84^

**Figure 4:**
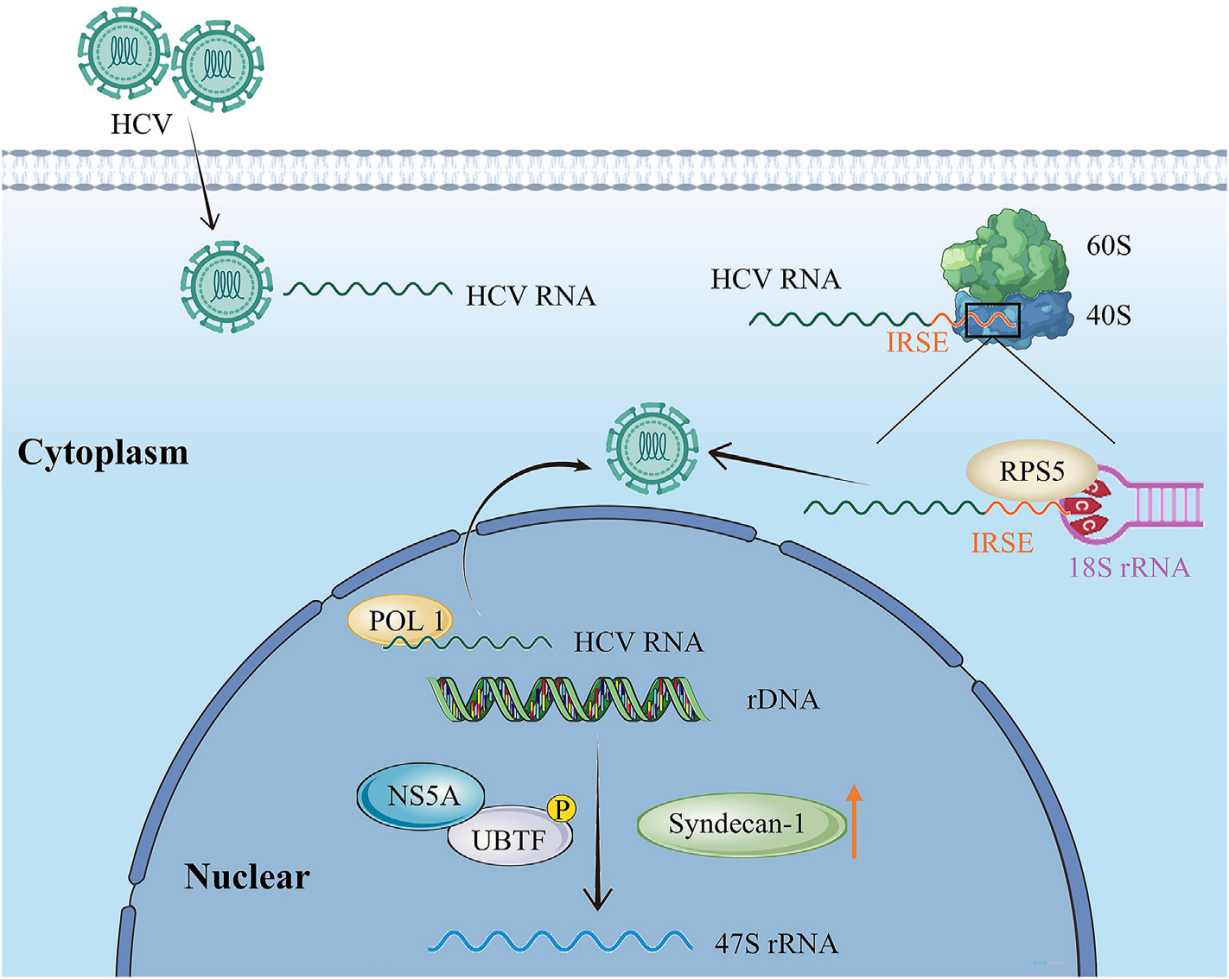
Schematic of ribosome biogenesis and hepatitis C virus (HCV). HCV enters the human body, its internal ribosome entry site (IRSE) fragment interacts with the 40S small subunit, and its RNA can also directly interact with RNA polymerase I (Pol I) to promote its protein translation. In addition, nonstructural protein 5A (NS5A) transduces signals into the nucleoplasm through upstream binding transcription factor (UBTF) hyperphosphorylation, and Syndecan-1 also acts on rRNA transcriptional activation, thereby increasing ribosome biogenesis.

HCV internal ribosome entry site (IRES) contacts the backbone and bases of the CCC triplet in the 18S ribosomal RNA expansion segment 7. These contacts provide interplay between IRES domain II and the AUG codon close to ribosomal protein S5, which causes a rearrangement of the 18S rRNA structure. This activates 40S ribosomes for subsequent translation initiation steps.^85^ HCV nonstructural protein 5A (NS5A) can transduce signals into the nucleoplasm via upstream binding transcription factor (UBTF) hyperphosphorylation leading to rRNA transcription activation, resulting in increased ribosome biogenesis, and contributing to the development of liver fibrosis and cirrhosis.^86^ HCV-infected livers produce extremely large amounts of syndecan-1. Syndecan-1 aggravates liver inflammation by activating rDNA translation to promote ribosome biogenesis.^87^ RNA Pol I-transcribed HCV genome RNA replicates produce an infectious virus and lead to severe hepatic steatosis in transgenic mice.^88^ Inhibiting ribosome biogenesis reduces HCV synthesis, thereby inhibiting liver deterioration (Fig. 4).

## Ribosome biogenesis and NAFLD

**Figure 5:**
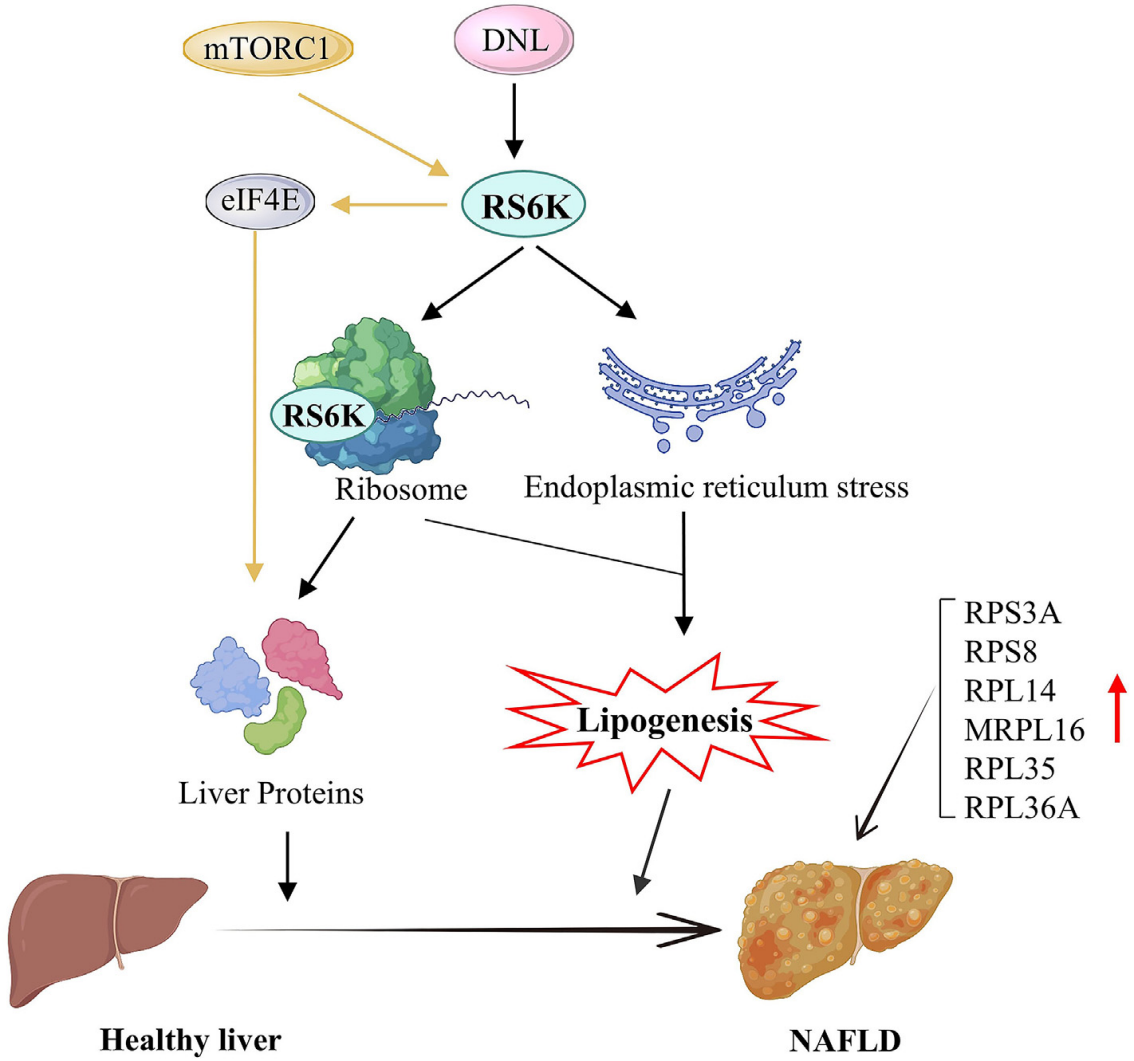
Ribosomal proteins accelerate lipogenesis, leading to nonalcoholic fatty liver disease (NAFLD). mTORC1 pathway enhances ribosomal protein S6 kinase (RS6K), resulting in activating eukaryotic initiation factor 4E (eIF4E), leading to the activation of liver protein translation leading to NAFLD. Fructose-induced *de novo* lipogenesis (DNL) involves RS6K-driven augmentation of hepatic protein synthesis and translation overdrive coupled with endoplasmic reticulum stress contributes to lipogenesis, leading to NAFLD.

NAFLD is a kind of metabolic comprehensive disease caused by excessive accumulation of fat in the liver without excessive alcohol consumption.^89^ Ribosomal protein family genes (RPL35, RPS3A, RPS8, and MRPL16) are identified as immune-cell-related biomarkers of NAFLD by bioinformatics and experimental analyses.^90^ Differentially expressed genes between the livers of the normal group and the NAFLD or non-alcoholic steatohepatitis groups were enriched in ribosomes (RPL36A, RPL14, *etc*.).^91^ The PI3K/mTOR pathway enhances ribosomal protein S6 kinase (RS6K), resulting in activating eukaryotic initiation factor 4E (eIF4E), leading to the activation of 5 cap-dependent protein translation leading to NAFLD.^92^ Fructose-induced *de novo* lipogenesis involves RS6K-driven augmentation of hepatic protein synthesis, a major contributor to hepatic steatosis in NAFLD. RS6K-driven translation overdrive coupled with endoplasmic reticulum stress contributes to lipogenesis, and RS6K inhibition is a therapeutic strategy to counter fructose-induced hepatic steatosis in NAFLD.^93^ These results might contribute to understanding the NAFLD mechanism, conducting experimental research, and designing clinical practices (Fig. 5).

## Ribosome biogenesis and liver fibrosis

**Figure 6:**
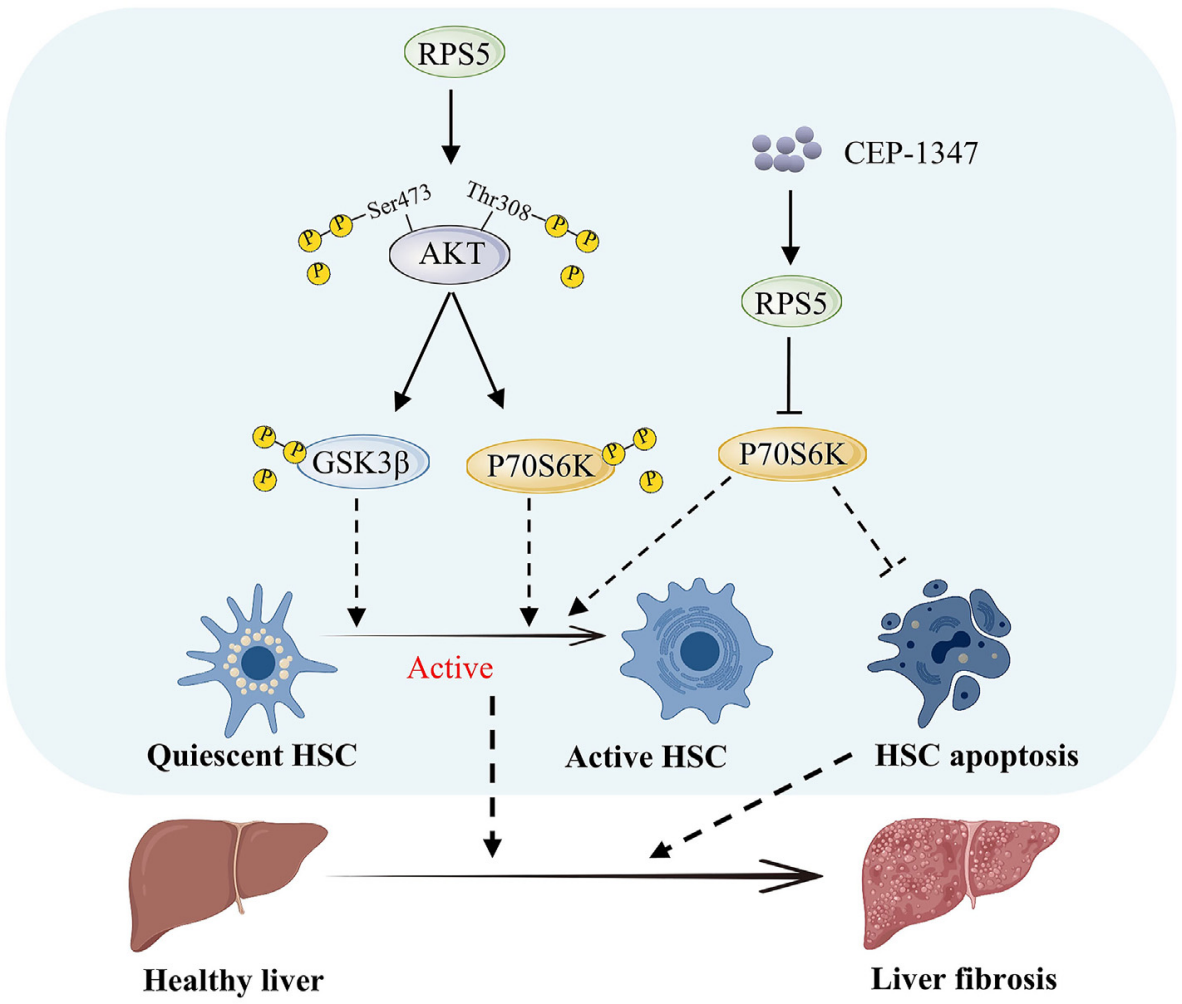
Ribosomal proteins accelerate the progression of liver fibrosis. Ribosomal protein S5 (RPS5) is overexpressed, it leads to the dephosphorylation of Akt at Ser473 and Thr308, dephosphorylating glycogen synthase kinase 3β (GSK3β) or P70S6K, ultimately accelerating the activation of stationary hepatic stellate cells (HSCs). CEP-1347 targets RPS5 to inhibit the expression of p70S6K and the activation of HSCs, thus accelerating the progression of liver fibrosis.

Liver fibrosis refers to the diffuse excessive deposition and abnormal distribution of the liver extracellular matrix, which is the pathological repair response of the liver to chronic injury. It is a key step in the development of various chronic liver diseases to cirrhosis and an important link affecting the prognosis of chronic liver diseases.^94^ Increased ribosome biogenesis levels can activate hepatic stellate cells, thereby aggravating fibrosis (Fig. 6).

An interesting finding is that RPS5 can prevent hepatic stellate cell activation. When RPS5 is overexpressed, it leads to the dephosphorylation of Akt at Ser473 and Thr308, dephosphorylating GSK3β or P70S6K. This cascade of events ultimately attenuates liver fibrosis induced by dimethylnitamine or biliary duct ligation.^95^^,^^96^ Remarkably, CEP-1347, a therapeutic agent for chronic liver disease and liver fibrosis, targets RPS5 to inhibit the expression of p70S6K and the activation of hepatic stellate cells, effectively blocking the progression of liver fibrosis.^97^ Additionally, blocking the activity of p70S6K directly induces apoptosis in hepatic stellate cells, thereby inhibiting fibrosis. Indirectly, it also reduces liver damage and inflammation.^98^ Reducing the expression of ribosome biogenesis-related proteins levels inhibits hepatic stellate cell activation and alleviates liver fibrosis.

## Ribosome biogenesis and liver cirrhosis

Liver cirrhosis is a progressive chronic liver disease caused by a variety of causes and is characterized histologically by diffuse hepatocyte necrosis, abnormal hepatocyte regeneration, angiogenesis, massive proliferation of fibrous tissue, and pseudo-lobule formation.^99^

**Figure 7:**
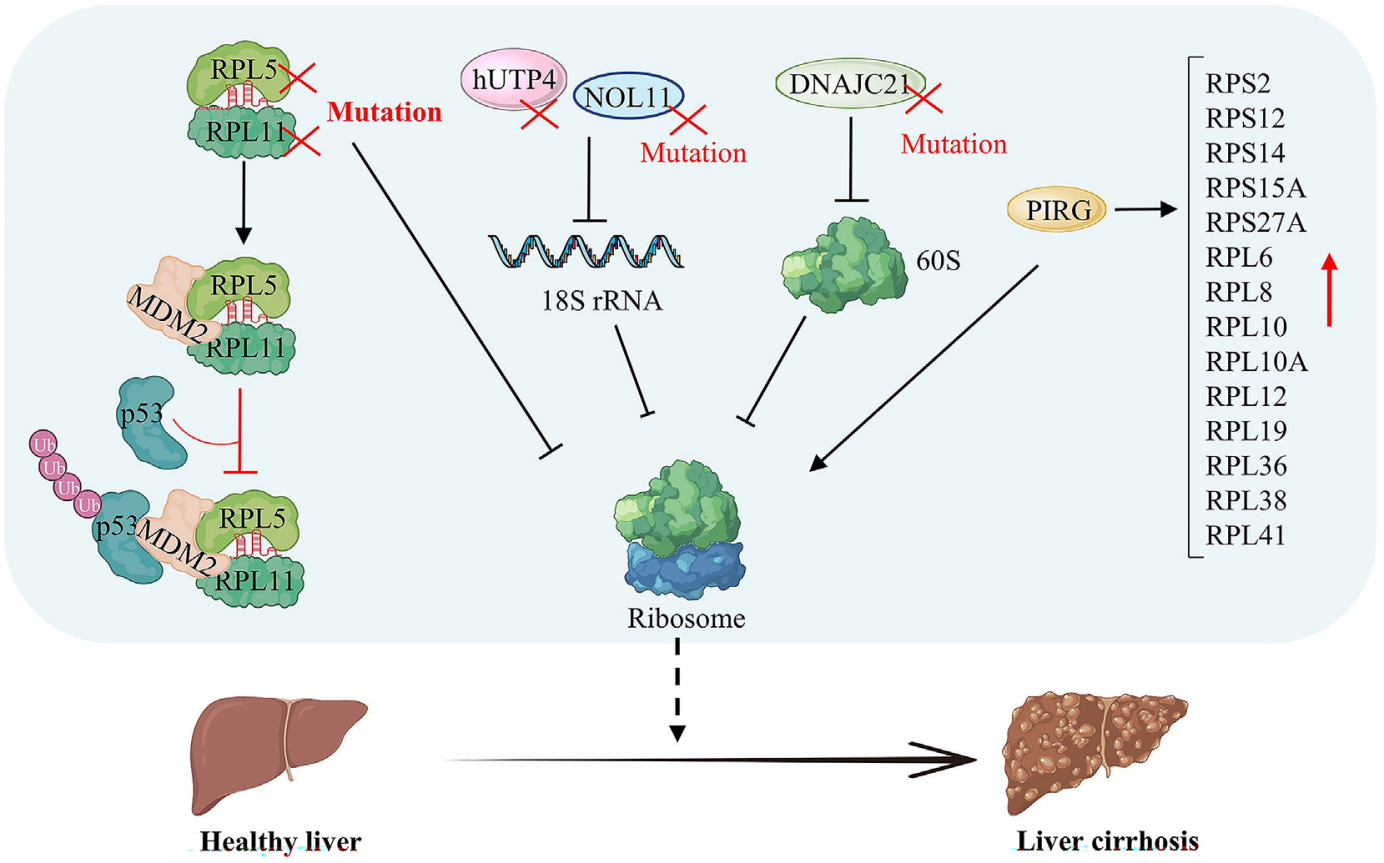
Ribosomal protein mutations lead to reduced ribosome biogenesis and promote liver cirrhosis. Mutated ribosomal protein L5 (RPL5) and ribosomal protein L11 (RPL11) bind to the proto-oncogene MDM2 and inhibit the ubiquitination of p53. Mutations in the ribosome assembly factors hUTP4 (human U three protein 4)/Cirhin and NOL11 (nucleolar protein 11) inhibit the synthesis of 18S rRNA, eventually inhibiting ribosome biogenesis leading to cirrhosis. Mutations in the factor DNAJC21 (DNAJ heat shock protein family (Hsp40) member C21), which synthesizes the 60S ribosome subunit, hinder ribosome biogenesis and lead to cirrhosis.

Ribosomal protein mutations reduce ribosome biogenesis and promote liver cirrhosis progression (Fig. 7). Ribosomal biogenesis is a crucial process required for cell growth and division. Mutated RPL5 and RPL11 bind to the proto-oncogene MDM2 and inhibit the ubiquitination of p53. It is a congenital ribosomopathy that manifests with symptoms such as liver cirrhosis.^100^^,^^101^ Mutations in the ribosome assembly factors hUTP4 (human U three protein 4)/Cirhin and NOL11 (nucleolar protein 11), responsible for synthesizing 18S rRNA, have been found to cause biliary cirrhosis in North American Indian children.^102^^,^^103^ Another protein, DNAJ heat shock protein family (Hsp40) member C21 (DNAJC21), plays a role in synthesizing the 60S ribosomal subunit. Mutations in DNAJC21 hinder ribosome biogenesis and can lead to hereditary bone marrow failure syndrome, often accompanied by liver cirrhosis.^104^ Polymeric immunoglobulin receptor (PIGR) is highly expressed in human liver cirrhotic tissues. RNA sequencing analysis revealed a significant up-regulation of various RPs (RPL10, RPL10A, RPL12, RPL19, RPL36, RPL38, RPL41, RPL6, RPL8, RPS12, RPS14, RPS15A, RPS2, and RPS27A) in the PIGR overexpression group. This suggests that PIGR may stimulate the ribosome pathway and contribute to the development of cirrhosis.^105^

## Ribosome biogenesis and liver cancer

**Figure 8:**
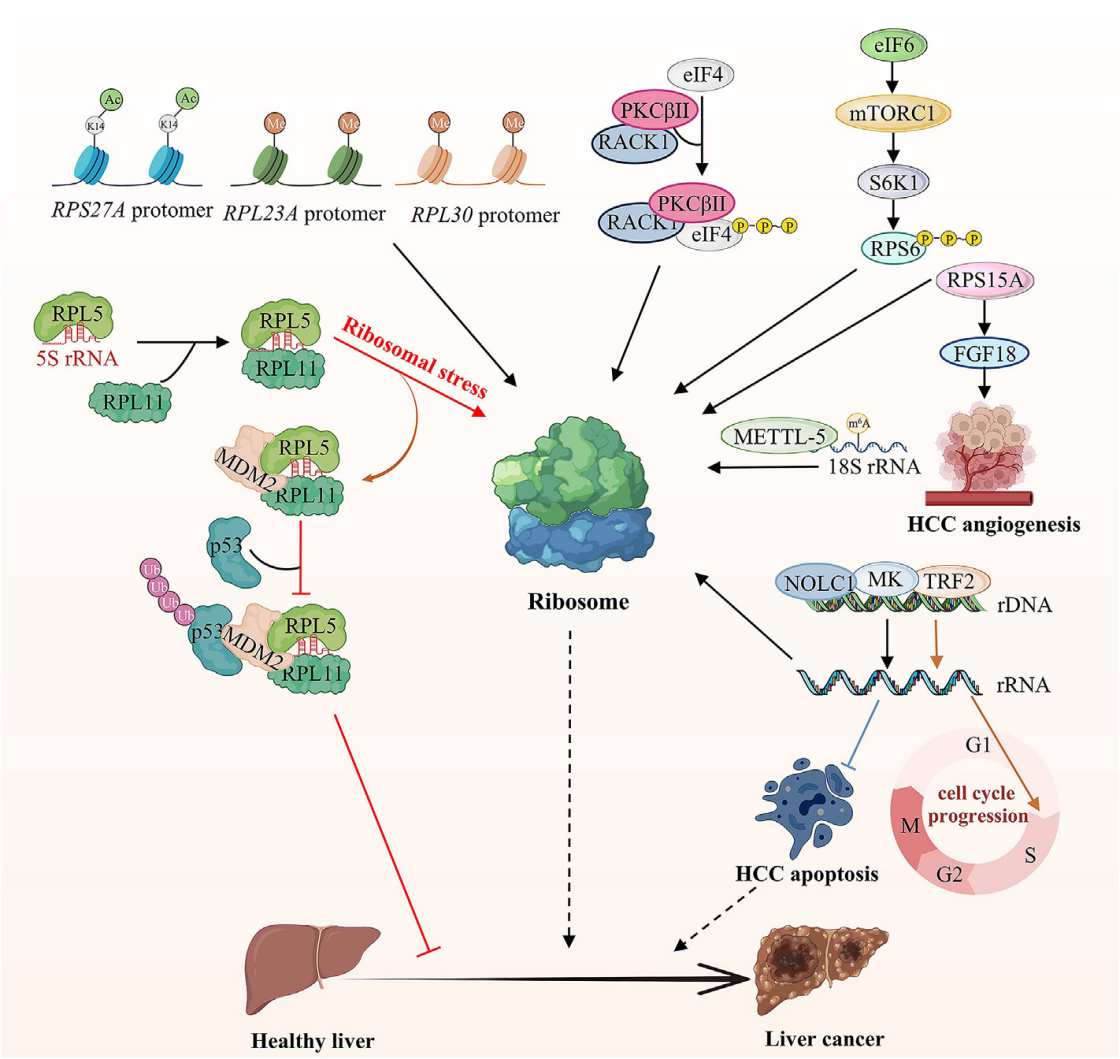
Schematic of ribosome biogenesis and liver cancer. RP-Mdm2-p53 pathway reduces ribosome biogenesis, stabilizes the p53 gene, and inhibits liver cancer. H3K14 acetylation of ribosomal protein S27A (RPS27A) and DNA methylation of ribosomal protein L23A (RPL23A) and ribosomal protein L30 (RPL30) induces hepatocellular carcinoma (HCC). Eukaryotic initiation factor 4/6 (eIF4 and eIF6) promote liver cancer by promoting ribosome biogenesis. Ribosomal protein S15A (RPS15A) promotes tumor angiogenesis via enhancing Wnt/beta-catenin-induced fibroblast growth factor 18 (FGF18) expression in HCC. Methyltransferase 5 (METTL5)-mediated 18S rRNA m^6^A modification promotes 80S ribosome assembly and induces HCC. Telomere repeat binding factor 2 (TRF2) binds to rDNA, promotes rRNA transcription in HCC, and attenuates nucleolar stress-induced HCC cell cycle arrest. Midkine (MK) and nucleolar and coiled-body phosphoprotein 1 (NOLC1) increase their expression and enhance 47S pre-rRNA transcription to protect HepG2 from apoptosis and promote cancer growth.

Abnormal increases in nucleolar size and number caused by dysregulation of ribosome biogenesis have emerged as a hallmark in most cancers (Fig. 8).^106^, ^107^, ^108^ Ribosome biogenesis leads to an enlargement of nucleolar organizing regions (NORs) and using AgNOR staining to label nucleoli is emerging as a diagnostic marker for cancer cells.^109^^,^^110^ Large amounts of AgNORs in hepatocytes are associated with an increased risk of HCC in chronic liver disease.^111^ In a prospective study of 64 HCC biopsy samples belonging to different stages (stages I to IVB), Shiro et al classified AgNORs into T1 (large nucleoli with clear edges) and T2 (thin black nucleoli without clear edges).^112^ Furthermore, this study demonstrates that HCC with smaller and/or irregular T1-NOR combined with high T2-NOR scores have the potential to be more aggressive. The number of AgNORs of HCC was significantly higher than those of normal liver and cirrhotic liver.^113^ This description corroborates the findings that AgNOR may be a useful indicator for assessing disease progression in HCC. In addition, exogenous stresses such as mechanical and spatial cues (collagen deposition and extracellular matrix remodeling) in the cirrhotic liver microenvironment are also factors that influence nucleolar number and morphology.^114^ Therefore, the AgNOR Quantitative Committee of the European Society of Pathology declared it the gold standard. Therefore, the quantitative distribution of nucleolar-associated AgNORs in interphase is a reliable predictor of disease progression and clinical outcome in multiple types of tumors, especially HCC. The nucleolus serves as the primary site of ribosome biogenesis, and enhanced ribosome biogenesis is also considered a predictor of cancer progression.^115^

Aberrant RNA modifications can lead to dysregulated gene expression and cancer. RP-Mdm2-p53 pathway reduces ribosome biogenesis, stabilizes the p53 gene, and inhibits liver regeneration without affecting nucleolar integrity. Upon ribosome biogenesis stress, such as silencing of RNA Pol I, impaired Ribosome biogenesis checkpoint (IRBC) complex (RPL5, RPL11, and 5S rRNA) becomes increasingly directly tethered to MDM2, resulting in suppressed ubiquitination-mediated p53 degradation. Increased rRNA synthesis also reduces the p53-mediated response to cytotoxic stress.^116^, ^117^, ^118^

Changes in ribosomal protein and rRNA expression contribute to liver cancer. The increased expression of various RPs, such as RPS8, RPL12, RPL23a, RPL27, and RPL30, has been associated with liver tumor growth.^119^^,^^120^ Analysis of databases such as TCCA and GEO reveals that RPL19 is highly expressed in human HCC tissues, with significant enrichment of the cell cycle pathway. This suggests that RPL19 may play a crucial role in promoting tumor progression and could be a promising biomarker and therapeutic target for accurate diagnosis and treatment of HCC.^121^ RPL30 reacts with the sera of HCC patients, and antibodies against RPL30 can be used as tumor markers.^122^ Furthermore, RPL36 is involved in the early development of HCC and can serve as an independent and potential prognostic marker for HCC resection.^123^^,^^124^

The RPS27A promoter is labeled with H3K14 acetylation, and acetyl-CoA synthetase 2 (ACSS2-S2) increases the expression of RPs by promoting acetylation, thereby enabling the carcinogenesis of HCC.^125^ DNA methylation of RPL23A and RPL30 induces HCC.^126^

Ribosomal RACK1 (receptor for activated C kinase 1) coupled with protein kinase CβII (PKCβII) to promote the phosphorylation of eIF4E, which led to HCC growth and chemotherapy resistance.^127^^,^^128^ Eukaryotic translation initiation factor 6 (eIF6) acts on 60S ribosomal subunit maturation. Inhibition of eIF6 activity effectively reduces lipid accumulation and hepatocellular growth.^129^ eIF6 enables the proliferation and invasion of human HCC by activating mTOR-related signaling pathways.^130^ The mTOR signal transduction pathway activates the protein kinase RS6K, which phosphorylates the RPS6 protein and activates ribosome biogenesis.^131^ Methyltransferase 5 (METTL5)-mediated 18S rRNA m^6^A modification accelerates 80S ribosome assembly and the translation of mRNAs involved in fatty acid metabolism, promoting fatty acid metabolism, oncogenic transformation, and tumor growth.^132^^,^^133^ RPS15A promotes tumor angiogenesis via enhancing Wnt/β-catenin-induced fibroblast growth factor 18 (FGF18) expression through the Wnt/β-catenin pathway in HCC.^134^^,^^135^

The increased ribosome biogenesis during G1/S arrest further worsens the epithelial-to-mesenchymal transition process and the development of metastatic cancer.^115^^,^^136^ For instance, in H4-II-E-C3 rat liver cancer cells, an increase in the appropriate complement of ribosomal RNA can exacerbate the transition from the G1 to S phase, promoting the growth and metastasis of liver cancer.^137^ Telomere repeat binding factor 2 (TRF2) in the nucleolus binds to rDNA and promotes rRNA transcription in HCC. Overexpression of TRF2 attenuates nucleolar stress-induced HCC cell cycle arrest.^138^^,^^139^ Midkine is mainly localized in the nucleolus, and increasing its expression enhances 47S pre-rRNA transcription. This protects HepG2 from apoptosis and promotes cancer growth.^140^ Nucleolar and coiled-body phosphoprotein 1 (NOLC1) expression is increased in HCC tissue, and its reduction inhibits rRNA processing, proliferation of HCC cells, and tumor growth.^141^^,^^142^

snoRNAs are highly conserved, stable non-coding RNAs involved in post-transcriptional modification of RNA and ribosome biogenesis.^143^, ^144^, ^145^, ^146^ They can act as oncogenes or tumor suppressors in HCC through multiple mechanisms. Further research on snoRNAs is crucial for the prevention and treatment of HCC.^147^

## Ribosome biogenesis and liver cancer therapy

Aberrant ribosome biogenesis is increasingly recognized as a viable therapeutic target for various tumors. Tumor cells produce more ribosomes than normal cells, and inhibiting ribosome biogenesis makes tumor cells more susceptible than normal cells. The pharmacological inhibition of ribosome biogenesis triggers the nucleolar stress response.^148^, ^149^, ^150^

**Figure 9:**
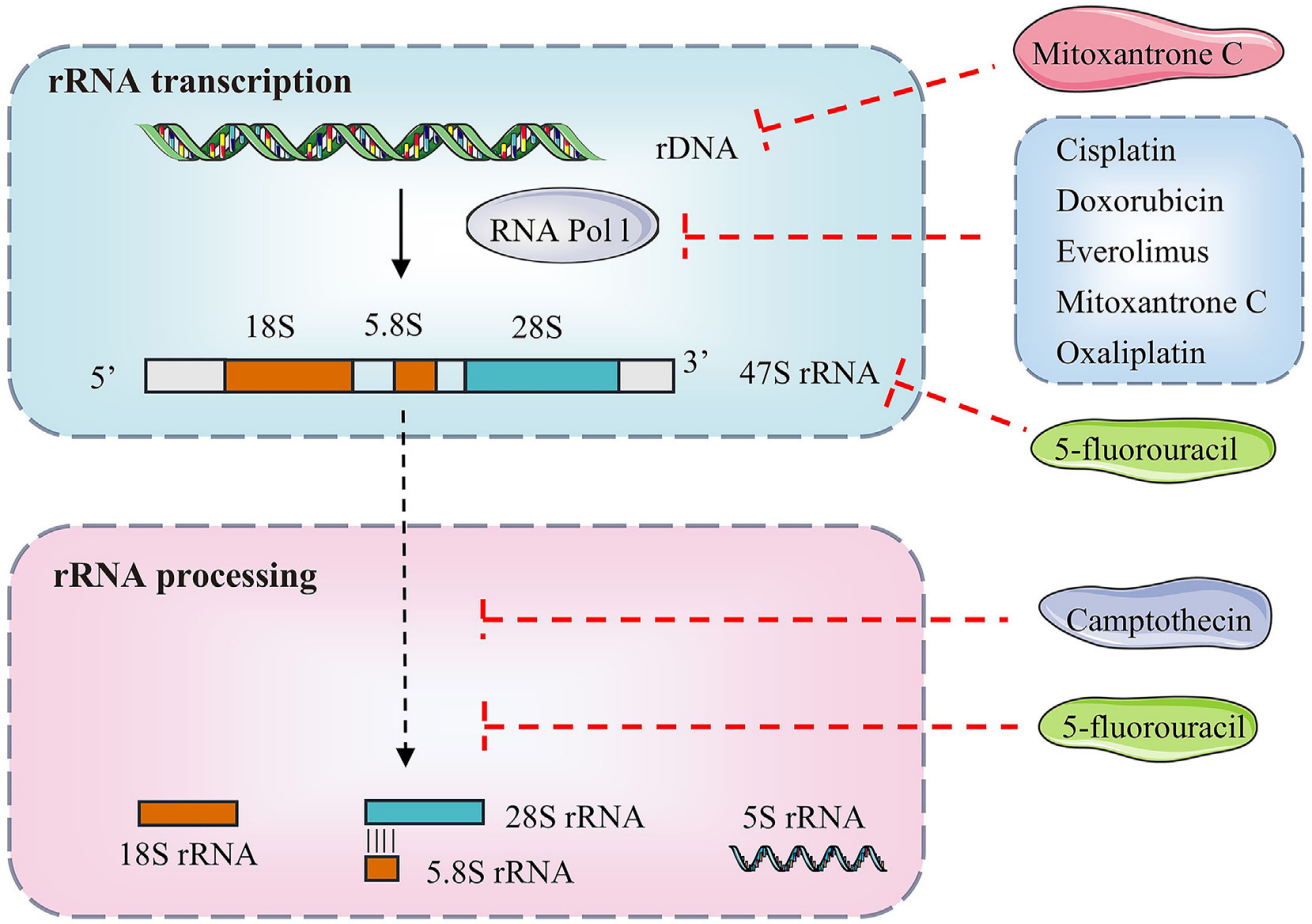
Effects of different anti-liver cancer drugs on rRNA transcription and processing. Cisplatin, doxorubicin, everolimus, and oxaliplatin inhibit the transcription of rDNA into rRNA by inhibiting RNA polymerase I (pol I). 5-Fluorouracil inhibits thymidylate synthetase, binds to 47S rRNA, and inhibits the post-processing of rRNA. Camptothecin inhibits the early processing of rRNA.

In tumor transformation, the abnormal p53 pathway stimulates nucleoli function, causing nucleoli enlargement.^151^^,^^152^ Drug discovery efforts targeting ribosome biogenesis have demonstrated some effectiveness. Table 1 shows the mechanisms of drugs used to treat liver cancer, adverse effects on the liver, and clinical trials. Traditional chemotherapeutic drugs like oxaliplatin, doxorubicin, and cisplatin have been shown to inhibit ribosome biogenesis at the rRNA transcription level. Oxaliplatin induces alkylation crosslinking of DNA bases and inhibits Pol I, resulting in early nucleolar destruction by suppressing rRNA synthesis and causing nucleophosmin 1 (NPM1) relocation, ultimately leading to extensive nucleolar recombination.^153^^,^^154^ Mild reversible increases in liver function indicators alanine transaminase and aspartate aminotransferase can occur in patients who have received platinum compounds.^155^ Cisplatin induces alkylation crosslinking of DNA bases and inhibits Pol I. Cisplatin has improved early liver cancer, while oxaliplatin and doxorubicin have been used for advanced HCC^156^, ^157^, ^158^ Cisplatin has been associated with low-rate serum enzyme elevations during liver cancer therapy. These elevations are usually mild, self-limited, and asymptomatic, rarely requiring dose modification.^159^ Doxorubicin inserts into DNA, inhibiting topoisomerase Ⅱ and Pol I.^160^ Serum aminotransferase levels are elevated upon doxorubicin therapy, but the elevations are generally asymptomatic and transient and would resolve even with continuation of therapy.^161^ Camptothecin inhibits topoisomerase Ⅰ and acts on the early processing of rRNA.^162^ It can cause elevations in serum aminotransferase levels.^163^ 5-fluorouracil inhibits thymidylate synthetase, binds with 47S rRNA, and inhibits the post-processing of rRNA.^164^ 5-fluorouracil is typically combined with leucovorin (folinic acid) which also inhibits thymidylate synthase, thus enhancing the effects of fluorouracil. Current indications for fluorouracil with leucovorin include palliative therapy for advanced liver cancer. 5-Fluorouracil has been used as a continuous hepatic arterial infusion to manage hepatic metastases from colorectal and other cancers.^165^ 5-fluorouracil is extensively metabolized in the liver via the microsomal enzyme system, and the production of a toxic intermediate may trigger liver injury.^166^^,^^167^ Everolimus is an inhibitor of cell proliferation and an immunosuppressive agent (mTOR inhibitor) that inhibits RNA Pol I.^168^ Medication of everolimus elevates serum enzyme levels, but the abnormalities are usually mild, asymptomatic, and self-limiting, rarely requiring dose modification or discontinuation (Fig. 9).^169^

**Table 1:** Clinical trials targeting liver cancer and ribosome biogenesis.

Drug	Mechanism	Adverse reaction	Phase	Trial identifier
5-Fluorouracil	Binds to 47S rRNA, and inhibits the post-processing of rRNA	5-Fluorouracil is extensively metabolized in the liver via the microsomal enzyme system, and the production of a toxic intermediate may trigger liver injury	Approved for use	Approved for use
Camptothecin	Acts on the early processing of rRNA	Serum aminotransferase elevations	Phase 3	NCT02755311
Cisplatin	Induces alkylation crosslinking of DNA bases and inhibits polymerase I	Mild, self-limited serum enzyme elevations	Phase 3	NCT00109954
Doxorubicin	Inserts into rDNA	Serum aminotransferase elevations are generally asymptomatic and transient	Phase 3	NCT01655693
Everolimus	An mTOR inhibitor that inhibits RNA polymerase I	Serum aminotransferase elevations are generally asymptomatic and transient	Phase 4	NCT02081755
Oxaliplatin	Inhibits RNA polymerase I and causes early nucleolar destruction by suppressing rRNA synthesis	Reversibly increased hepatic alanine transaminase and aspartate aminotransferase levels	Phase 2	NCT00052364

The combined use of drugs targeting multiple ribosome biogenesis has the potential to produce improved therapeutic effects. For instance, combining oxaliplatin and fluorouracil has shown a better therapeutic response in liver cancer.^170^, ^171^, ^172^ CX-5461 is a specific inhibitor of ribosome biogenesis that does not cause genotoxicity. It selectively inhibits the function of RNA Pol I, thereby achieving ribosome biogenesis.^173^^,^^174^ Currently, CX-5461 is being used to treat breast cancer^175^ and ovarian cancer,^176^, ^177^, ^178^, ^179^ and its effectiveness in liver cancer will be further investigated.

## Discussion

Ribosome biogenesis is an evolutionarily conserved protein synthesis machine, and ribosome biogenesis ensures that ribosomes are present in the body.^180^ Ribosome biogenesis includes rDNA transcription, cleavage of pre-rRNA, modification to form mature rRNA, RP synthesis and translocation into the nucleus, and assembly of rRNA into large and small subunits.^181^ Therefore, different steps of ribosome biogenesis can be targeted to achieve the treatment of specific diseases. We review ribosome biogenesis and mechanisms of liver regeneration, HCV, NAFLD, liver fibrosis, cirrhosis, and liver cancer that cause liver disease by affecting human liver ribosome biogenesis. The review aims to provide scientists with a new idea for understanding how ribosome-related liver disease occurs and provide a theoretical basis for developing a drug to treat ribosome diseases.

HCV needs to rely on human ribosomes to complete replication.^182^ Studies of HCV IRES have shown its direct interaction with the ribosome, which induces a conformational change of the ribosome.^183^ Therefore, HCV infection and the progression of liver disease are closely related to ribosome biogenesis. For several decades, ribavirin was combined with pegylated interferon alpha (Peg–IFN-α) as the standard of care for treating chronic HCV infections.^184^ Targeting the site where HCV binds to ribosomes after infecting the liver may prevent HCV from replication in the liver, thereby inhibiting HCV.

Ribosomes temporarily increase to repair the damaged liver and promote liver regeneration after acute liver injury. When there is an excessive and abnormal increase in ribosomes in the liver, it will gradually deteriorate. Inhibition of RNA Pol I transcription triggers nucleolar stress, resulting in the translocation of RPs from the nucleolus to the nucleoplasm, where proteins such as RPL5 and RPL11 bind to MDM2, triggering its dissociation and thereby stimulating p53.^118^ Therefore, the concept of inhibiting RNA Pol I for cancer therapeutics attracted investigators to design specific inhibitors to target RNA Pol I, with the expectation that normal cells would be spared because they are much less dependent on RNA Pol I transcription activity than cancer cells.

At present, there is no clear effective method for treating liver fibrosis, liver cirrhosis, and liver cancer. Pol I regulates rDNA transcription to generate rRNA, which is the rate-limiting step in ribosome biogenesis and plays a central role in cancer progression. Abnormally increased Pol I activity will destroy the ribosomal function of the nucleolus, causing uncontrolled ribosome synthesis and leading to malignant cell proliferation. Therefore, Pol I is an excellent target for selectively inhibiting cancer cell growth. Several Pol I transcription inhibitors have been developed for cancer treatment, including CX-5461 and BMH-21. CX-5461 was the first selective and orally available inhibitor of RNA Pol I transcription.^185^ CX-5461 is the first Pol I inhibitor to complete phase I clinical trials. It inhibits Pol I transcription mainly by competing with the pre-initiation complex protein SL1 for the rDNA promoter.^186^ BMH-21 is the newly discovered pol I inhibitor that inhibits transcription initiation and elongation by binding to rDNA. BMH-21 can significantly reduce the viability of HCC cells *in vitro* and the growth of HCC *in vivo* but has little effect on liver function or body weight.^187^ Oxaliplatin, doxorubicin, cisplatin, camptothecin, 5-fluorouracil, *etc*. have been used to treat liver cancer. In clinical, drugs can be combined to increase their efficacy or reduce their toxic and side effects.

Several drugs targeting ribosome biogenesis are also in clinical trials to treat other cancers. CX-5461 inhibits the progression of advanced solid cancer by binding to and stabilizing the G4 DNA structure (NCT04890613).^174^ CX-3543 binds to the G4 sequence and disrupts the interaction of the rDNA G4 structure with nucleolin, thereby inhibiting RNA Pol I function and inducing apoptosis in cancer cells. It has been used in clinical trials of advanced solid tumors (NCT00955786), lymphomas neuroendocrine tumors (NCT00780663), carcinoid cancer advanced solid tumors (NCT00955292), and lymphoma B-cell chronic lymphocytic leukemia (NCT00485966).^188^ Camptothecin treats sarcoma by inhibiting topoisomerase I, regulates early rRNA processing and has entered phase III clinical trials (NCT00354744).

Abnormal increase in nucleoli has been considered as a preliminary diagnostic indicator of liver cancer, but whether it can be used as an indicator of other liver diseases, such as NAFLD, liver fibrosis, and cirrhosis, remains to be further verified.

## Conclusions

In the liver, ribosome biogenesis is a complex process that involves multiple steps, from rRNA synthesis to ribosome assembly. Any errors in these steps can result in malignant transformation of liver cells and abnormal cell phenotype. Reduced ribosome biogenesis can lead to low-proliferative phenotypes such as cell cycle arrest, senescence, or apoptosis. On the other hand, increased ribosome biogenesis promotes liver regeneration, but excessive increase can lead to hepatocarcinogenesis. The nucleolus acts as a target of cancer signaling and an upstream regulator of pathways critical for average cell growth and function. The dynamic nucleolar proteome regulates cell function by controlling protein nucleolar localization and transport. Disturbances in normal nucleolar function and structure can disrupt ribosome biogenesis and contribute to liver disease.

Combining drugs targeting ribosome biogenesis with ribosome-related factors may enhance therapeutic effects in treating liver diseases. The drug direction can be designed for protein and rRNA synthesis involved in ribosome biogenesis or other steps, such as assembly. This research has the potential to discover new therapeutic strategies for liver disease.

Conducting a systematic and comprehensive analysis of mRNA translation, protein localization, and molecular changes upstream and downstream of RPs or rRNA alterations will contribute to developing more targeted ribosome therapy for liver diseases.

## Funding

This work was supported by the 10.13039/501100001809National Natural Science Foundation of China (No. 82272421), the Changzhou's 14th Five-Year Plan Project to Train High-Level Health Professionals (Jiangsu, China) (No. 2022CZLJ027), Changzhou Special Program for the Introduction of Foreign Talents (No. CQ20240052) and Open project of Jiangsu Provincial Key Laboratory of Key Laboratory of Laboratory Medicine (No. JSKLM-Z-2024-001, JSKLM-Z-2024-002).

## CRediT authorship contribution statement

**Wei Luo:** Writing – review & editing, Writing – original draft. **Jing Zhou:** Investigation. **Yongmin Yan:** Writing – review & editing. **Xuezhong Xu:** Writing – review & editing.

## Conflict of interests

The authors have no competing interests to declare.
